# Unilateral Molar Distalization: A Nonextraction Therapy

**DOI:** 10.1155/2012/846319

**Published:** 2012-12-25

**Authors:** M. Bhanu Prasad, S. Sreevalli

**Affiliations:** Department of Orthodontics and Dentofacial Orthopedics, Dr. Sudha and Nageswararao Siddhartha Institute of Dental Sciences, Chinoutuplli, Gannavaram, Krishna District, Andhra Pradesh 521 286, India

## Abstract

In the recent years, nonextraction treatment approaches and noncompliance therapies have become more popular in the correction of space discrepancies. One of the conventional approaches for space gaining in the arches without patient compliance is done by using certain extra oral appliances or intraoral appliance. The greatest advantage of certain appliances like fixed functional and molar distalization appliances is that they minimize the dependence on patient cooperation. Molar distalization appliances like pendulum appliance which distalizes the molar rapidly without the need of head gear can be used in patients as a unilateral space gaining procedure due to buccal segment crowding.

## 1. Introduction

Pendulum appliance is one of the molar distalization appliance used intraorally. This was introduced by Hilgers in 1992. The basic appliance consists of nance palatal component with rests that are welded to premolar and molar bands. The distalization mechanism consists of bilateral helical spring made out of titanium molybdenum alloy. Unlike Jones jig, it does not have any coil springs; instead, it has 0.032 inches TMA springs which deliver a continuous force against the maxillary first molar producing 200 to 250 gms of force in a swimming arc movement from the midline, hence the name pendulum [1, 2]. Usually this appliance is given in the maxillary arch than in the mandibular arch due to the bone pattern. This intraoral design includes two elements: the active component which distalizes the maxillary molars and the anchorage unit that compensates for the reactionary forces. The anchorage unit is a combination of dental anchorage and soft tissue rests or absolute different skeletal anchorage systems (Implants). An ideal intraoral molar distalizer should meet the following criteria: minimal patient compliance, straight profile, mild loss of anterior anchorage (as evidenced by the axial proclination of the incisors), distalization of molars bodily, and minimal chair side time for placement and reactivations. Among the distalizing methods introduced, the Hilgers Pendulum Appliance seems to satisfy these requirements. Even this device, however, can produce unwanted tipping of the maxillary molars during distalization [3].

Common instance of space requirement is to relieve crowding or aligning of impacted tooth. Indication for molar distalization is the presence of good soft tissue profile, mild-to-moderate space requirement (borderline case), and finally the absence of the third molar. The side effects of these appliances are the mild proclination of the anterior teeth and the opening up of the mandibular plane angle. Protrusion of anterior can be counteracted by using class-II elastics [4]. Therefore case selection according to growth pattern (horizontal/vertical grower) is very important before we use these appliances.

## 2. Case Report

A 17-year-old female reported to the Orthodontic Department with a chief complaint of irregularly placed upper and lower front teeth. On examination she had mild skeletal class III malocclusion with angle's class-I molar relation on both sides. Overretained “C” and “E” are present on the right side of the maxillary arch. Impacted canine is present on the right side with an anterior deep bite. Soft tissue profile indicated a straight profile with competent lips. Treatment involved the extraction of overretained deciduous teeth and 32 (lower left instanding lateral incisor) which is lingually erupting. Later aligning the palatally impacted canine into the arch and settling the occlusion with preadjusted edgewise appliance (0.022 ROTH) is done. 

**Figure 1:**
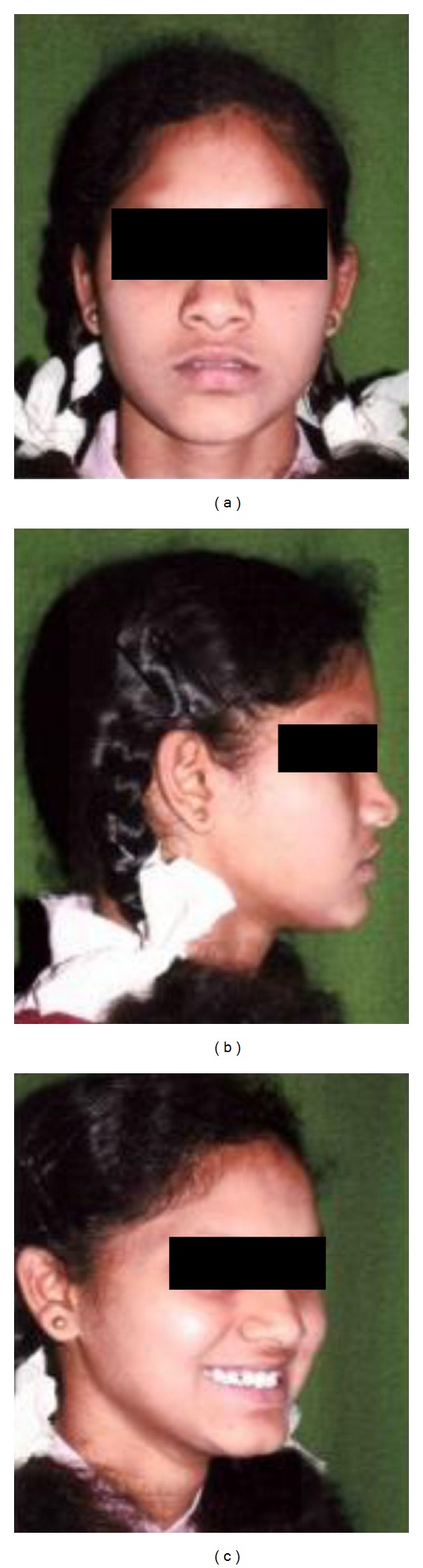
Profile of the patient showing straight profile with normodivergent growth pattern.

Soft tissue profile indicated a straight profile with competent lips in Figure 1.

**Figure 2:**
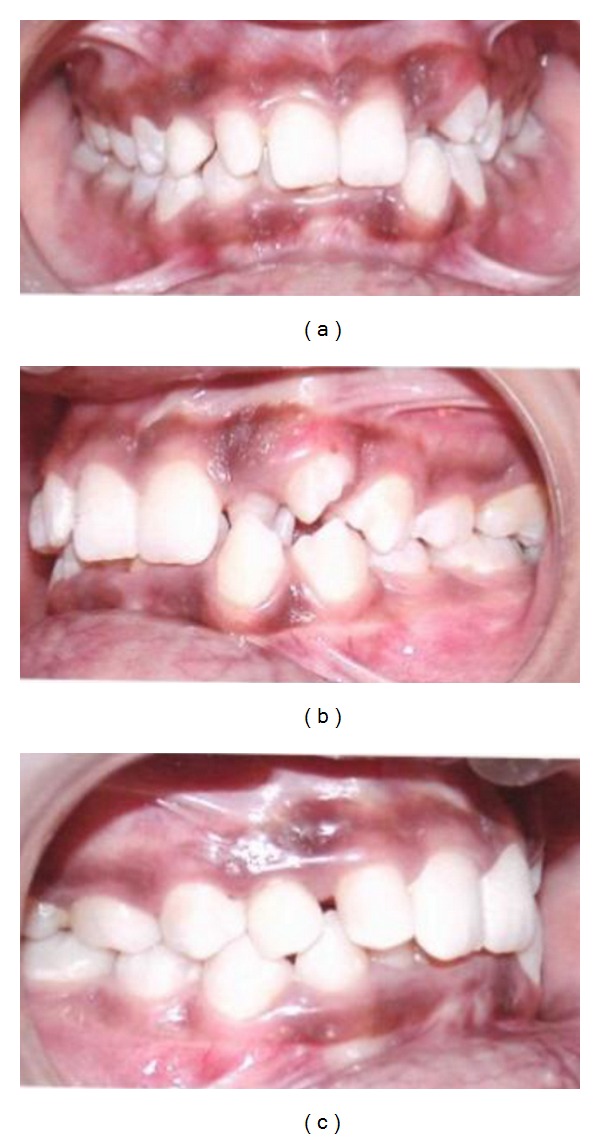
Anterior deep bite with crowding in upper and lower arches.

Patient exhibited an anterior deep bite with crowding in the lower anterior region seen in Figure 2. 

**Figure 3:**
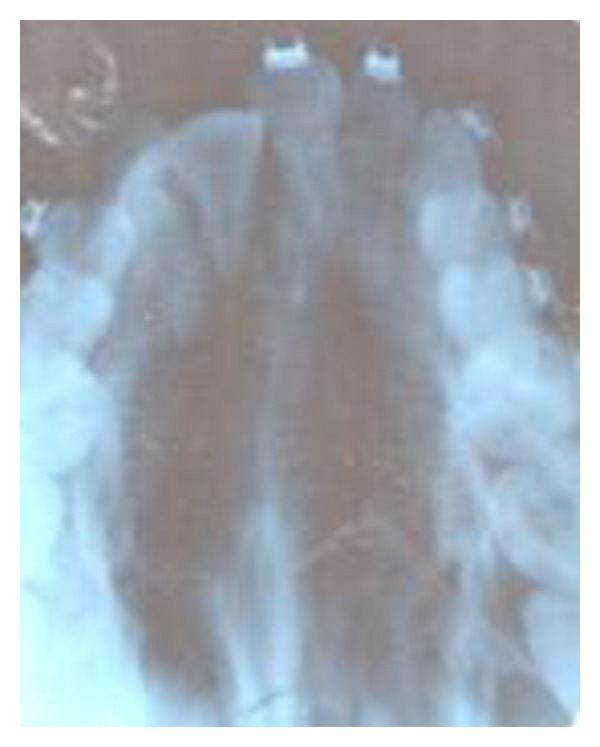
X-ray showing favorably impacted canine.

The occlusal X-ray film in Figure 3 revealed favourably an impacted canine for alignment. 

**Figure 4:**
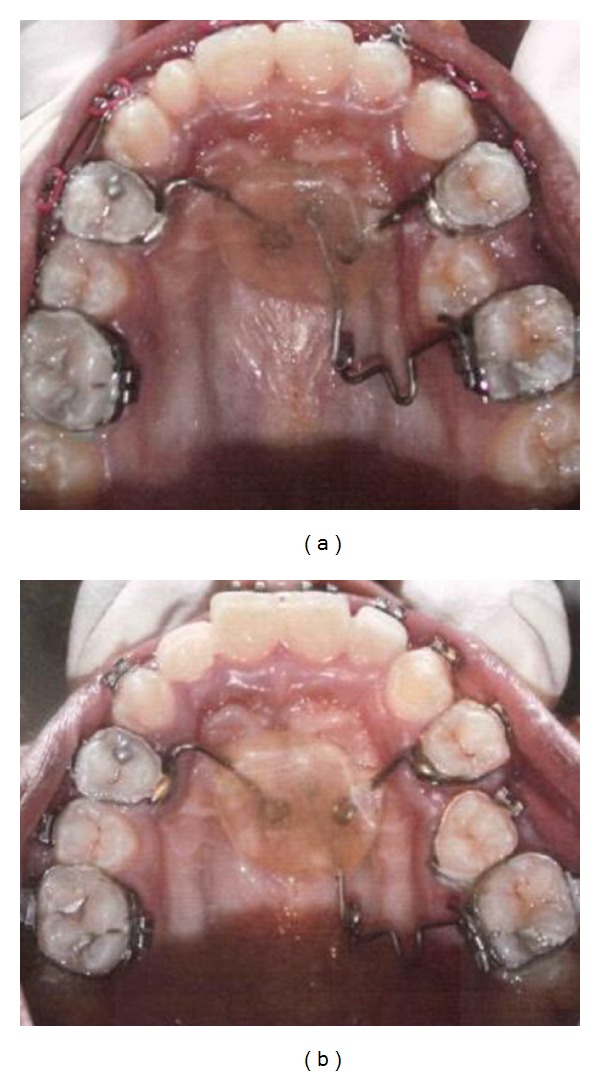
Unilateral molar distalization appliance used to align the impacted canine and 2nd premolar on right side.

Extraction of overretained deciduous teeth is done before the exposure of canine. Mucoperiosteal flap is raised and canine crown is exposed. Bracket bonded on the crown and elastic chain are tied from 13 to the 0.018SS arch wire. Unilateral molar distalization is done on the right side to create space for the canine as well as the 2nd premolar which is developing crossbite a after aligning canine, Figure 4.

**Figure 5:**
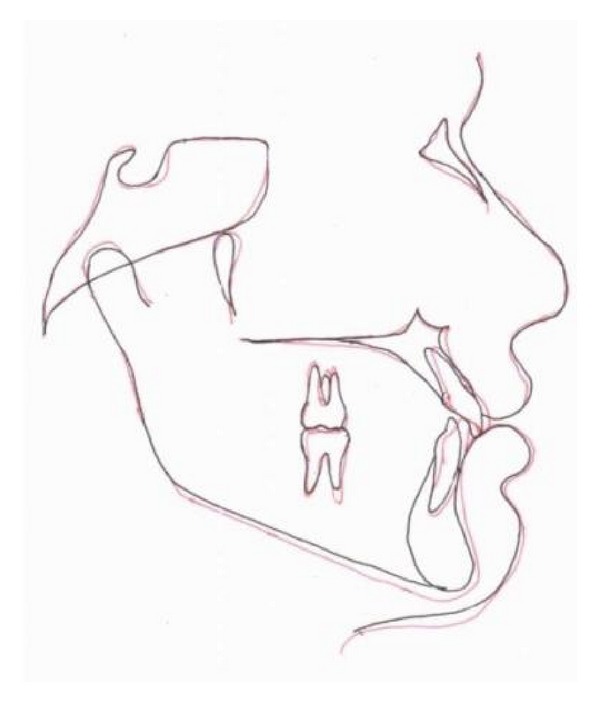
Superimpositions showing maxillary incisor proclination, molar distalization, and mild opening of the mandibular plane angle.

Cephalometric superimpositions showed mild proclination of maxillary anterior and extrusion of upper molar to some extent as shown in Figure 5.

**Figure 6:**
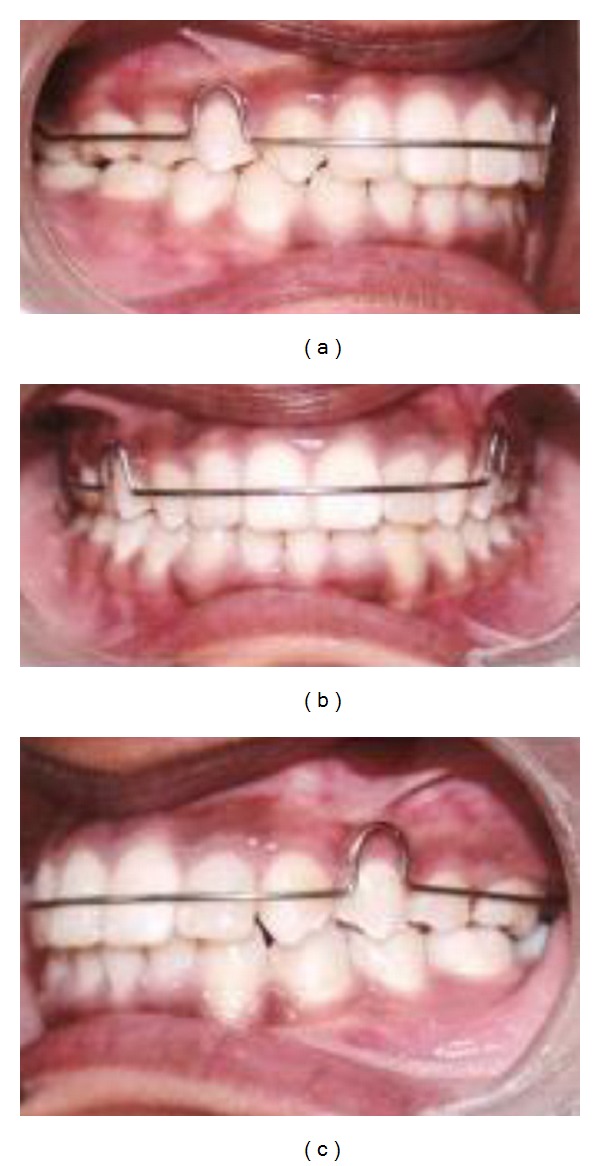
Postretention with Hawley's retainer and lower fixed retainer at the end of leveling and aligning.

After the leveling and aligning of upper and lower arches, debonding is done after treatment retention followed Hawley retainer in the upper arch and fixed retainer in the lower arch seen in Figure 6. 

## 3. Discussion

The noncompliance intraoral molar distalization method has been an excellent compromise for patients who are unwilling to wear headgear. There is always a marked individual variation in patient's response to these appliances in terms of anchorage loss and skeletal effects. For guided molar distalization, TMA wire of 0.032 is used. The use of this beta titanium wire allowed to provide constant distal force near to the centre of the resistance of molar, thus reducing the moment of force [5, 6]. In this case, distalization of molar occurred with the minimum amount of anchor loss. It may be due to the support taken from a wide acrylic button and the inclusion molar on other side along with two premolar rests. 

In the saggital plane, molar distalization occurred at the expense of the mild proclination of the maxillary anterior teeth due to reciprocal mesial force, thus causing anchorage loss which is favourable in this case as the patient is having deepbite and straight profile [7]. In the vertical plane, this appliance extruded the maxillary molar, thus increasing the mandibular plane angle to a mild degree which caused the downward and backward rotation of the mandible. The clockwise rotation of mandible in this patient reduced her Class-III tendency [8]. In the transverse plane, the rotation occurred is very less when compared to the crossbites that occurred due to the usage of unilateral headgears as mentioned by Siatkowski's [7, 9]. By giving a mild toe-in bend, the molar rotation is reduced in this case [10]. In this case, satisfactory molar distalization by 2 mm has occurred. Minor inflammation of palatal mucosa was determined after the removal of the appliance. This is prevented with the maintenance of a proper oral hygiene. 

To conclude, pendulum appliance acts as an effective molar distalizer in space discrepancy problems present in the buccal segment.

## References

[1] Hilgers JJ (1992). The pendulum appliance for Class II non-compliance therapy. *Journal of Clinical Orthodontics*.

[2] Ghosh J, Nanda RS (1996). Evaluation of an intraoral maxillary molar distalization technique. *American Journal of Orthodontics and Dentofacial Orthopedics*.

[3] Scuzzo G, Pisani F, Takemoto K (1999). Maxillary molar distalization with a modified pendulum appliance. *Journal of Clinical Orthodontics*.

[4] Keles A, Sayinsu K (2000). A new approach in maxillary molar distalization: intraoral bodily molar distalizer. *American Journal of Orthodontics and Dentofacial Orthopedics*.

[5] Keles A (2002). Unilateral distalization of a maxillary molar with sliding mechanics: a case report. *Journal of Orthodontics*.

[6] Reiner TJ (1992). Modified Nance appliance for unilateral molar distalization. *Journal of Clinical Orthodontics*.

[7] Siatkowski RE (1997). *,Asymmetric Headgear in Nanda(Red) Biomechanics in Orthodontics*.

[8] Kinzinger GSM, Fritz UB, Sander FG, Diedrich PR (2004). Efficiency of a pendulum appliance for molar distalization related to second and third molar eruption stage. *American Journal of Orthodontics and Dentofacial Orthopedics*.

[9] Cangialosi TJ, Melstrell ME, Leung MA, Ko JY (1988). A cephalometric appraisal of edgewise Class II nonextraction treatment with extraoral force. *American Journal of Orthodontics and Dentofacial Orthopedics*.

[10] Bussick TJ, McNamara JA (2000). Dentoalveolar and skeletal changes associated with the pendulum appliance. *American Journal of Orthodontics and Dentofacial Orthopedics*.

